# More than a digestive disorder: Perceived stigma in inflammatory bowel disease patients

**DOI:** 10.1371/journal.pone.0352696

**Published:** 2026-06-26

**Authors:** Neslihan Güneş Aydemir, Ali Çağrı Oral, Tuğçe Taşar Yıldırım

**Affiliations:** 1 Department of Gastroenterology, Elazığ Fethi Sekin City Hospital, Elazig, Turkey; 2 Department of Internal Medicine, Elazığ Fethi Sekin City Hospital, Elazig, Turkey; Instituto Nacional de Ciencias Medicas y Nutricion Salvador Zubiran, MEXICO

## Abstract

**Background:**

Inflammatory bowel disease (IBD) is a chronic condition characterized by symptoms that are often difficult to conceal and may expose patients to negative social reactions. Beyond physical burden, stigma represents an important but underrecognized psychosocial challenge in IBD, potentially affecting social functioning and disease management. To determine the prevalence of perceived stigma among patients with IBD and to evaluate its association with disease subtype, sociodemographic characteristics, clinical features, and self-esteem.

**Methods:**

A total of 146 patients with IBD under regular follow-up were included in this cross-sectional study. Perceived stigma was assessed using ten disease-specific yes/no items reflecting real-life stigma-related experiences. Sociodemographic data, clinical characteristics, and self-esteem levels measured by the Rosenberg Self-Esteem Scale were recorded. Patients were classified as experiencing stigma if they endorsed at least one stigma item.

**Results:**

Overall, 63.0% of patients reported at least one stigma-related experience. The most frequently reported manifestations were social avoidance and discomfort in public settings, while overt discrimination was less common. The distribution of stigma-related experiences was largely similar between Crohn’s disease and ulcerative colitis patients. Individuals reporting stigma were significantly younger and had a younger age at diagnosis compared to those without stigma. No significant differences were observed with respect to gender, marital status, education, employment, income, disease subtype, surgical history, or comorbidities. Patients experiencing stigma had significantly lower self-esteem scores than those without stigma.

**Conclusion:**

Perceived stigma is common among patients with IBD, particularly among younger individuals, and is more often expressed as social discomfort and avoidance rather than explicit discrimination. These findings highlight stigma as an important psychosocial dimension of IBD that warrants greater clinical attention. Integrating psychosocial support into routine care may help mitigate stigma-related consequences and improve overall disease management. Longitudinal studies are needed to clarify the long-term impact of stigma on disease outcomes.

## Introduction

Stigma associated with illness refers to negative attitudes and discriminatory behavior toward people with a specific illness [1,2]. In the context of Inflammatory Bowel Disease (IBD), stigma is strongly linked to symptoms that are difficult to conceal, including fecal urgency, diarrhea, gas, abdominal pain, fear of accidents, preoccupation with toilet access, weight changes, and surgical scars. Research indicates that many individuals with IBD perceive themselves as “different” and “stigmatized,” frequently leading to social avoidance and withdrawal [3,4]. The literature generally describes three types of stigma. Perceived stigma refers to the individual’s belief that surrounding people judge, feel disgusted by, or disapprove of them due to their illness; the patient may feel this even without overt discriminatory behavior from others. Enacted stigma involves direct, concrete experiences of discrimination, exclusion, belittlement, or ridicule within environments such as work, school, family, or the healthcare system. Lastly, internalized stigma (or self-stigma) occurs when the individual incorporates these negative societal judgments into their own self-concept, leading them to view themselves as shameful, worthless, or “flawed.” [5–7]. Chronic diseases such as IBD are often accompanied not only by physical symptoms but also by psychological and social consequences. Stigma is one of the most significant psychosocial challenges faced by individuals with chronic illnesses, as it can lead to feelings of shame, social isolation, and decreased adherence to treatment. Previous studies have shown that patients with visible or socially misunderstood diseases, such as mental illness, HIV, or obesity, frequently experience stigmatization; however, research on disease-related stigma in IBD remains limited, particularly in countries where cultural and societal perceptions of gastrointestinal disorders differ [2,8–10]. Moreover, existing stigma scales have generally been developed for other chronic conditions and may not adequately capture the unique experiences of IBD patients. Understanding the prevalence and determinants of stigma in this group is therefore crucial for improving patient-centered care, enhancing treatment adherence, and addressing the psychosocial dimensions of chronic disease management.

The present study aimed to determine the prevalence of perceived stigma among patients with IBD under regular follow-up and to examine whether the experience of stigma varied according to disease subtype or sociodemographic and clinical characteristics.

## Materials and methods

### Study design and participants

This descriptive cross-sectional study was conducted between 01/12/2025 and 10/01/2026. Participant recruitment started on 01/12/2025 and ended on 10/01/2026 among patients with a confirmed diagnosis of IBD who were under regular follow-up at the Gastroenterology Department of a tertiary care hospital. Data were collected using a structured online questionnaire developed via Google Forms, which included four sections: (1) sociodemographic information, (2) disease-related characteristics, (3) the Rosenberg Self-Esteem Scale (RSES), and (4) ten stigma-related yes/no questions developed for this study. The survey link was sent to eligible participants via email using the hospital’s patient database. Since all participants were registered follow-up patients, their contact information was available through hospital records. Prior to participation, written informed consent was obtained electronically from all participants. A snowball sampling method was applied: initial participants were invited via email, and they were encouraged to share the survey link with other IBD patients within their patient network. Only those who voluntarily responded were included in the analysis. The inclusion criteria were: being 18 years or older, having a confirmed diagnosis of Crohn’s disease or ulcerative colitis, being literate, having access to the internet, and agreeing to participate voluntarily. Exclusion criteria included: having a cognitive or psychiatric condition that could affect comprehension, having an incomplete survey (more than 10% missing responses), or being newly diagnosed within less than three months. Ethical approval for the study was obtained from the Elazığ Fethi Sekin City Hospital Non-Interventional Research Ethics Committee (Decision No: 2025/19–5; Date: 20 November 2025). Electronic informed consent was obtained from all participants before participation in the study.

The primary outcome was the prevalence of perceived stigma. The sample size for a single proportion was calculated with a 95% confidence level using n_0_ = Z^2^p(1 − p)/d^2^ [11,12]. Assuming p = 0.36 [13] and a margin of error d = 0.07, the required sample was 113; allowing for ~15% non-response, the target minimum sample size was set at ~130 participants.

### Rosenberg’s self-esteem scale (RSES)

The RSES is a 10-item instrument that evaluates individuals’ overall self-worth by measuring positive and negative self-perceptions. It uses a 4-point Likert scale ranging from strongly agree (3 points) to strongly disagree (0 points), with total scores ranging from 0 to 30; higher scores indicate greater self-esteem. In the scoring process, items 3, 5, 8, 9, and 10 are reverse-coded [14,15]. The Turkish adapted version of the RSES has been widely validated and is commonly used to assess self-esteem and its relationship with psychosocial factors, including stigma, among clinical populations [2,14–16]. In this study, the RSES was used to assess self-esteem levels and to explore its association with perceived stigma in patients with IBD.

### Assessment of stigma

To assess stigma in patients with IBD, ten questions were prepared by the research team. These questions were developed based on a review of previous studies on disease-related stigma [2,17–22] and through consultations with two gastroenterologists experienced in clinical management of IBD. Since there is currently no validated disease-specific stigma scale available for IBD in Turkey, these questions were designed to evaluate patients’ perceptions of social, emotional, and behavioral aspects of stigma. This was not a standardized scale but a set of simple items with dichotomous (Yes/No) response options. Each question explicitly probed a concrete, lived situation using a yes/no format (e.g., “Do you hesitate to tell others that you have IBD?”, “Do you avoid social activities because of your IBD symptoms?”). This wording captures personal disclosure, social perception, and behavioral avoidance rather than abstract attitudes, allowing a direct estimate of the proportion of patients who actually experience each aspect of stigma. The evaluation was based on the distribution of participants who answered “Yes” to each question. Moreover, stigma was operationally defined as responding “Yes” to at least one of the ten disease-specific stigma questions, indicating the presence of perceived stigma related to IBD. Participants who did not endorse any item (“No” to all questions) were classified as having no perceived stigma. This definition was used to estimate the overall prevalence of stigma among IBD patients.

Although the ten IBD stigma-related questions were not developed as a standardized psychometric scale, their internal consistency was examined to assess the reliability and coherence of the questions. The Kuder-Richardson Formula 20 (KR-20) coefficient was calculated. The internal consistency of these ten questions was acceptable, with a KR-20 value of 0.83

### Statistical analysis

Statistical analyses were performed with IBM SPSS 26.0 (SPSS Inc., Chicago, USA). Categorical data were presented as numbers and percentages, while continuous variables were expressed as mean ± standard deviation (SD) and median (min-max). The normal distribution of continuous variables was assessed using both visual (histograms and probability plots) and analytical methods (Kolmogorov-Smirnov and Shapiro-Wilk tests). They were found not to be normally distributed. Comparisons between two independent groups (Crohn’s disease vs. ulcerative colitis) were conducted using the Mann-Whitney U test for continuous variables and the Chi-square test (or Fisher’s exact test, where applicable) for categorical variables. A heatmap visualization was generated to illustrate the distribution of “Yes” responses to the ten IBD stigma-related questions. The heatmap was created using Python (version 3.11) with the Seaborn and Matplotlib libraries. The percentage values of affirmative responses for each item were arranged in a three-column matrix (total sample, Crohn’s disease, and ulcerative colitis), and color intensity was used to represent the relative frequency of “Yes” responses. Darker shades correspond to higher proportions, facilitating visual comparison across the two IBD subgroups.

Variables that showed statistically significant differences between patients with and without perceived stigma in univariable analyses (p < 0.05) were included in a binary logistic regression model to evaluate factors independently associated with perceived stigma. Regression results were presented as regression coefficient (B), odds ratio (OR), 95% confidence interval (95% CI), and p value.

A p-value of <0.05 was considered statistically significant.

## Results

A total of 146 patients with IBD were included in the study. The mean age of the participants was 43.7 ± 15.3 years, and the median age at diagnosis was 35.5 years (range: 12.0–70.0). The mean disease duration was 7.4 ± 7.1 years. More than half of the participants were female (52.1%) and married (59.6%). Regarding education, 37.0% had a university degree or higher, while 32.2% had completed primary or secondary school. Nearly half of the patients (43.9%) were not working, and 24.0% reported having no income. Most participants (80.8%) resided in city centers. Among disease-related characteristics, 33.6% of the patients had Crohn’s disease and 66.4% had ulcerative colitis. A total of 17.8% had a history of IBD-related surgery, and 14.6% had complications requiring surgical intervention. The vast majority (90.4%) regularly participated in health check-ups, and 42.5% had at least one additional comorbid disease (Table 1).

**Table 1:** Sociodemographic and clinical characteristics of participants (n = 146).

Variables	Values
**Age (year)**	
Mean±SD	43.7 ± 15.3
Median(min-max)	42.0 (18.0-76.0)
**Age at diagnosis (year)**	
Mean±SD	36.2 ± 13.9
Median(min-max)	35.5 (12.0-70.0)
**Duration of IBD (year)**	
Mean±SD	7.4 ± 7.1
Median(min-max)	5.0 (0.0-34.0)
**Gender, n (%)**	
Male	70 (47.9)
Female	76 (52.1)
**Marital status, n (%)**	
Married	87 (59.6)
Single	41 (28.1)
Divorced	12 (8.2)
Widowed	6 (4.1)
**Educational level, n (%)**	
Primary-Secondary school	47 (32.2)
High school	45 (30.8)
University and above	54 (37.0)
**Employment status, n (%)**	
Full-time	51 (34.9)
Part-time	6 (4.1)
Not working	64 (43.9)
Retired	25 (17.1)
**Income, n (%)**	
Low (below expenses)	39 (26.7)
Medium (income equal to expenses)	54 (37.0)
High (above expenses)	18 (12.3)
No income	35 (24.0)
**Permanent residence, n (%)**	
City center	118 (80.8)
District-village	28 (19.2)
**Type of IBD, n (%)**	
Crohn	49 (33.6)
Ulcerative colitis	97 (66.4)
**History of IBD surgery, n (%)**	26 (17.8)
Complications of requiring surgery, n (%)*	24 (16.4)
Abscess	6 (4.1)
Fistula	9 (6.2)
Intestinal obstruction	9 (6.2)
Stricture	8 (5.5)
**Participation in regular health check-ups, (%)**	132 (90.4)
**Another comorbid disease, n (%)**	62 (42.5)
**RSES Total score**	
Mean±SD	21.3 ± 4.6
Median(min-max)	21.0 (7.0-30.0)

SD: Standard deviation; * Some patients experienced more than one complication

When comparing patients with Crohn’s disease and ulcerative colitis, significant differences were found in educational level, history of IBD-related surgery, and surgical complications. Patients with ulcerative colitis had higher education levels (p = 0.019), while those with Crohn’s disease more frequently had a history of surgery (p < 0.001) and surgery-related complications (p = 0.036). Other variables showed no significant differences between the groups (Table 2).

**Table 2:** Sociodemographic and clinical characteristics of participants according to IBD type (n = 146).

Variables	Crohn n = 49	Ulcerative colitis n = 97	p
**Age (year)**			0.728*
Median(min-max)	43.0 (18.0-68.0)	42.0 (19.0-76.0)	
**Age at diagnosis (year)**			0.850*
Median(min-max)	35.0 (12.0-66.0)	36.0 (15.0-70.0)	
**Duration of IBD (year)**			0.505*
Mean±SD	6.9 ± 6.9	7.7 ± 7.1	
Median(min-max)	5.0 (0.0-34.0)	5.0 (0.0-30.0)	
**Gender, n (%)**			0.484**
Male	21 (42.9)	49 (50.5)	
Female	28 (57.1)	48 (49.5)	
**Marital status, n (%)**			0.068**
Married	22 (44.9)	65 (67.0)	
Single	19 (38.8)	22 (22.7)	
Divorced	6 (12.2)	6 (6.2)	
Widowed	2 (4.1)	4 (4.1)	
**Educational level, n (%)**			0.019**
Primary-Secondary school	15 (30.6)	32 (33.0)	
High school	22 (44.9)	23 (23.7)	
University and above	12 (24.5)	42 (43.3)	
**Employment status, n (%)**			0.246**
Working	15 (30.6)	42 (43.3)	
Not working	26 (53.1)	38 (39.2)	
Retired	8 (16.3)	17 (17.5)	
**Income, n (%)**			0.424**
Low (below expenses)	13 (26.5)	26 (26.8)	
Medium (income equal to expenses)	20 (40.8)	34 (35.1)	
High (above expenses)	3 (6.1)	15 (15.5)	
No income	13 (26.5)”	22 (26.8)	
**Permanent residence, n (%)**			1.000**
City center	40 (81.6)	78 (80.4)	
District-village	9 (18.4)	19 (19.6)	
**History of IBD surgery, n (%)**	17 (34.7)	9 (9.3)	<0.001**
**Complications of requiring surgery, n (%)**	13 (26.5)	11 (11.3)	0.036**
**Participation in regular health check-ups, (%)**	45 (91.8)	87 (89.7)	0.774**
**Another comorbid disease, n (%)**	21 (42.9)	41 (42.3)	1.000**
**RSES Total score** Median(min-max)	20.0 (12.0-27.0)	22.0 (7.0-30.0)	0.091*

*Mann-Whitney U test; **Chi-Square Test

**Fig 1:**
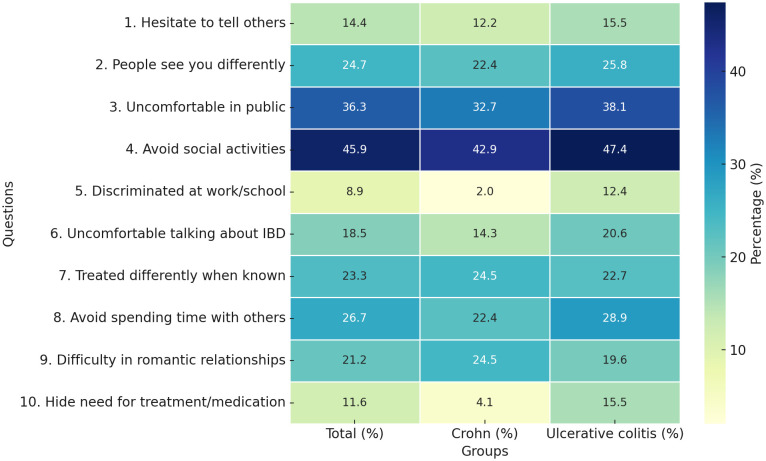
Distribution of “Yes” responses to IBD stigma-related questions.

In this study, stigma related to IBD was evaluated using ten concrete yes/no questions that reflected real-life situations patients may experience. When the responses were analyzed, it was found that 63.0% of all participants had answered “Yes” to at least one of the ten items, indicating the presence of perceived stigma. The most commonly endorsed stigma-related situations involved behavioral avoidance. Nearly half of the participants (45.9%) reported avoiding social activities because of their disease, and more than one-third (36.3%) stated that they felt uncomfortable in public due to IBD symptoms. Perceived social judgment was also frequent, as approximately one in four participants (24.7%) felt that people saw them differently because of their symptoms, and 23.3% said that others treated them differently after learning about their condition. Issues related to disclosure and communication were reported by 14–18% of patients; 14.4% hesitated to tell others about their diagnosis, and 18.5% felt uncomfortable talking about it. Difficulties in interpersonal and romantic relationships were present in around one-fifth of the participants, while structural forms of stigma, such as discrimination at work or school (8.9%) and concealing the need for medication or treatment (11.6%), were less frequently observed. Although ulcerative colitis patients tended to report slightly higher percentages for most social exposure items, the overall distribution pattern of stigma was largely comparable between disease subtypes (Table 3, Fig 1).

**Table 3:** Distribution of participants’ responses to IBD stigma-related questions (n = 146).

Questions related to IBD stigma, n (%)*	Total N = 146	Crohn n = 49	Ulcerative colitis n = 97
1. Do you hesitate to tell others that you have IBD?	21 (14.4)	6 (12.2)	15 (15.5)
2. Do you feel that people see you differently because of your IBD symptoms?	36 (24.7)	11 (22.4)	25 (25.8)
3. Do you feel uncomfortable in public because of your IBD?	53 (36.3)	16 (32.7)	37 (38.1)
4. Do you avoid social activities because of your IBD symptoms?	67 (45.9)	21 (42.9)	46 (47.4)
5. Do you think you are discriminated against at work or school because you have IBD?	13 (8.9)	1 (2.0)	12 (12.4)
6. Do you feel uncomfortable talking about your IBD?	27 (18.5)	7 (14.3)	20 (20.6)
7. Do you feel that people treat you differently when they learn that you have IBD?	34 (23.3)	12 (24.5)	22 (22.7)
8. Do you avoid spending time with friends or family because of your IBD symptoms?	39 (26.7)	11 (22.4)	28 (28.9)
9. Do you think you have difficulty in your romantic relationships because of your IBD?	31 (21.2)	12 (24.5)	19 (19.6)
10. Do you hide the fact that you need to receive treatment or take medication because of your IBD?	17 (11.6)	2 (4.1)	15 (15.5)
**At least one “Yes” response to stigma-related questions**	**92 (63.0)**	**30 (61.2)**	**62 (63.9)**

* The table presents the frequency (n) and percentage (%) of participants who responded ***“Yes”*** *to each stigma-related item.*

*The last row indicates participants who responded “Yes” to at least one of the ten stigma-related items, representing the overall presence of stigma (The comparison between Crohn’s disease and ulcerative colitis groups was performed using the Chi-square test (p = 0.891)*

Among the 146 patients with IBD, 92 (63.0%) reported experiencing stigma, defined as giving at least one “Yes” response to the disease-specific stigma questions, while 54 (37.0%) did not report any stigma experience. The comparison of sociodemographic and clinical characteristics between the two groups is presented in Table 4. Patients who experienced stigma were significantly younger than those without stigma (median age 38.0 vs. 49.0 years, p = 0.003) and were also diagnosed with IBD at a significantly younger age (median 30.0 vs. 41.5 years, p = 0.001). Although the duration of disease tended to be longer among patients with stigma (median 6.0 vs. 4.0 years), this difference did not reach statistical significance (p = 0.318). Gender, marital status, educational level, employment status, and income level did not differ significantly between those with and without stigma (all p > 0.05). Similarly, no significant differences were observed in place of residence, type of IBD (Crohn’s vs. ulcerative colitis), history of IBD surgery, complications requiring surgery, participation in regular health check-ups, or presence of another comorbid disease. However, a notable difference was observed in self-esteem scores. Patients who reported stigma had significantly lower RSES scores compared to those without stigma (median 20.5 vs. 23.0, p = 0.005) (Table 4).

**Table 4:** Comparison of sociodemographic and clinical characteristics between patients with and without perceived stigma in IBD.

Variables	No Stigma (n = 54)	Stigma Present* (n = 92)	*p*
**Age (year)**			0.003**
Median(min-max)	49.0 (19.0-76.0)	38.0 (18.0-70.0)	
**Age at diagnosis (year)** Median(min-max)	41.5 (15.0-70.0)	30.0 (12.0-64.0)	0.001**
**Duration of IBD (year)** Median(min-max)	4.0 (0.0-34.0)	6.0 (0.0-30.0)	0.318**
**Gender, n (%)**			0.703***
Male	27 (50.0)	43 (46.7)	
Female	27 (50.0)	49 (53.3)	
**Marital status, n (%)**			0.315***
Married	34 (63.0)	53 (57.6)	
Single	12 (22.2)	29 (31.5)	
Divorced	4 (7.4)	8 (8.7)	
Widowed	4 (7.4)	2 (2.2)	
**Educational level, n (%)**			0.524***
Primary-Secondary school	20 (37.0)	27 (29.3)	
High school	14 (26.0)	31 (33.7)	
University and above	20 (37.0)	34 (37.0)	
**Employment status, n (%)**			0.908***
Working	20 (37.0)	37 (40.2)	
Not working	24 (44.5)	40 (43.5)	
Retired	10 (18.5)	15 (16.3)	
**Income, n (%)**			0.246***
Low (below expenses)	11 (20.4)	28 (30.4)	
Medium (income equal to expenses)	21 (38.9)	33 (35.9)	
High (above expenses)	10 (18.5)	8 (8.7)	
No income	12 (22.2)	23 (25.0)	
**Permanent residence, n (%)**			1.000***
City center	44 (81.5)	74 (80.4)	
District-village	10 (18.5)	18 (19.6)	
**Type of IBD, n (%)**			0.891***
Crohn Ulcerative colitis	19 (35.2) 35 (64.8)	30 (32.6) 62 (67.4)	
**History of IBD surgery, n (%)**	7 (13.0)	19 (20.7)	0.343***
**Complications of requiring surgery, n (%)**	5 (9.3)	19 (20.7)	0.118***
**Participation in regular health check-ups, (%)**	8 (14.8)	6 (6.5)	0.144***
**Another comorbid disease, n (%)**	27 (50.0)	57 (62.0)	0.158***
**RSES Total score** Median(min-max)	23.0 (15.0-29.0)	20.5 (7.0-30.0)	0.005**

*Stigma was defined as having at least one “Yes” response to any of the ten disease-specific stigma-related questions;

**Mann-Whitney U test, ***Chi-Square Test

A binary logistic regression analysis was performed to evaluate factors independently associated with perceived stigma in patients with IBD. Variables showing statistically significant differences between patients with and without perceived stigma in univariable analyses in Table 4 were included in the model. Since age and age at diagnosis were closely related variables, only age was included in the final regression model to avoid collinearity. In addition, because there is no universally accepted cutoff value for the RSES, both age and RSES total score were analyzed as continuous variables.

The regression analysis demonstrated that younger age and lower RSES total scores were independently associated with perceived stigma. Specifically, increasing age was associated with a lower likelihood of reporting perceived stigma (B = −0.036, OR=0.964, 95% CI: 0.941–0.988, p = 0.003). Similarly, higher RSES total scores were associated with a lower likelihood of perceived stigma (B = −0.122, OR=0.885, 95% CI: 0.814–0.962, p = 0.004).

## Discussion

In our study, we investigated whether stigma is present among patients IBD. When evaluating responses, we found that approximately 60% of participants reported at least one stigma-related experience. This finding suggests that perceived stigma may be a common aspect in the lives of individuals managing IBD. Considering that stigma can affect emotional well-being, social participation and disease management, our results underline the importance of recognising and addressing the social dimension of this chronic condition. Previous research has consistently shown that individuals with IBD frequently experience stigma in various forms [5,13,21,22]. Stigma may emerge through social misunderstanding, negative self-perception, or hesitancy to disclose the disease, often irrespective of clinical disease activity [2,17,21,22]. Studies across different populations have highlighted that feelings of being judged, misunderstood, or treated differently are common among people living with IBD [5,21,22]. These findings collectively indicate that stigma is a pervasive psychosocial issue intertwined with IBD, reinforcing the importance of recognizing and addressing its impact on patients’ well-being and daily functioning [21].

Overall, our findings demonstrate that nearly two-thirds of individuals with IBD experience some form of stigma, primarily reflected in behavioral avoidance and social discomfort, rather than overt discrimination. These patterns of stigma in patients with IBD are consistent with previous literature emphasizing the psychosocial burden associated with chronic gastrointestinal conditions. Prior studies have shown that stigma is a multidimensional experience in IBD, often manifesting through behavioral avoidance, perceived social judgment, and reluctance to disclose the disease rather than overt discrimination. Such experiences can lead to increased social withdrawal, emotional distress, and reduced treatment adherence [1,13]. The high proportion of participants in our study who reported avoiding social activities or feeling uncomfortable in public mirrors findings from earlier research, where stigma was found to be prevalent across both active and inactive disease phases. This reinforces the notion that stigma in IBD is not solely driven by symptom severity but also by societal attitudes, self-perception, and communication barriers. Addressing these psychosocial aspects through patient education and supportive counseling could therefore play an essential role in improving overall disease management and quality of life.

In our study, younger patients were more likely to report perceived stigma, and younger age remained independently associated with stigma in the regression analysis. This finding suggests that younger individuals may be more vulnerable to the psychosocial effects of the disease. This age effect has also been observed in other chronic conditions and may be related to greater sensitivity to peer opinion and social image among younger adults [5]. Although ulcerative colitis patients reported slightly higher stigma levels than those with Crohn’s disease, the overall pattern was similar between groups, suggesting that stigma in IBD is not disease-type specific but rather influenced by individual and social factors, as supported by previous research in the field [6].

Although disease duration tended to be longer among those with stigma, this difference was not statistically significant. Importantly, patients who reported stigma had significantly lower RSES scores, and lower self-esteem remained independently associated with perceived stigma in the regression analysis. These findings support the negative relationship between stigma experiences and psychological well-being in patients with IBD. However, the present study did not use a validated disease-specific stigma scale and did not aim to develop one. Instead, stigma was explored through ten concrete IBD-related questions reflecting real-life social and emotional experiences associated with the disease. Although self-esteem and stigma may be conceptually related, they represent different psychosocial dimensions. The RSES evaluates general self-worth, whereas the questions used in this study specifically focused on disease-related social discomfort, disclosure concerns, behavioral avoidance, and perceived social judgment. Therefore, assessing IBD-related stigma may require more disease-specific and context-oriented evaluation tools. Future studies may focus on the development and psychometric validation of a standardized IBD-specific stigma scale.

Other sociodemographic and clinical factors such as gender, education, income, or disease type did not show significant associations. Guo et al. highlighted that perceived and internalized stigma are common in IBD and are strongly associated with negative emotions, social withdrawal, and reduced treatment adherence [5]. Similar to their observations, our participants frequently reported feeling uncomfortable in public or avoiding social activities due to IBD symptoms. In Guo’s review, concealment and selective disclosure were described as common coping strategies, often driven by fear of negative judgment or misunderstanding. This aligns with our finding that nearly one in five patients felt uneasy discussing their disease, and about 14% hesitated to tell others about their diagnosis. Furthermore, the tendency to hide medication use or treatment needs, reported by 11.6% of our participants, parallels Guo et al.’s observation that non-disclosure may lead to psychological stress and communication barriers [5,23].

Research has consistently demonstrated that stigma can significantly influence various aspects of disease management among patients with IBD. Perceived stigma has been associated with poorer treatment adherence, lower satisfaction with care, and worse clinical and psychological outcomes [5,13,17,21,22].

Taft and colleagues also found that internalized stigma was significantly associated with poorer psychological outcomes, including lower self-esteem, reduced self-efficacy, higher psychological distress, and decreased health-related quality of life [1,6,13]. These findings are consistent with the patterns observed in our study, where participants who reported stigma also described social withdrawal and difficulty communicating about their disease. Although our design did not include direct measures of psychological distress, the high prevalence of behavioral avoidance and discomfort suggests a similar psychosocial burden.

If left unaddressed, these feelings of shame or social withdrawal may exacerbate anxiety and depressive symptoms, further reduce quality of life, and create additional barriers to effective disease management. Understanding whether stigma is present in IBD and how it affects patients is clinically important. It helps clinicians recognize vulnerable patients who may need targeted psychosocial support or counseling, which can improve both their emotional well-being and their adherence to treatment.

This study has several strengths. It focused on an important but less studied topic, stigma in patients with IBD. The questions were developed specifically for IBD based on previous studies and expert opinions, which increased their clinical relevance. Including both Crohn’s disease and ulcerative colitis allowed comparison between subgroups. The internal consistency of the ten stigma questions was good (KR-20 = 0.83). Data collection through an online survey made participation easy and reduced interviewer bias. However, the study has some limitations. Its cross-sectional design prevents causal interpretation. The use of snowball sampling may limit generalizability. Furthermore, because data were collected through an online survey, individuals without internet access or sufficient digital literacy may have been underrepresented, potentially introducing selection bias toward younger or more digitally engaged participants. In addition, standardized disease activity indices such as the Harvey-Bradshaw Index or Mayo Score were not assessed in this study. Therefore, the potential effect of symptom severity or active disease on perceived stigma could not be evaluated. Despite these points, the study offers important preliminary data and highlights the need for further research on stigma in IBD.

## Conclusions

This study showed that stigma is common among patients with IBD, particularly among younger adults and individuals with lower self-esteem. About two-thirds of the participants reported at least one stigma-related experience. Feelings of social discomfort and avoidance were more common than open discrimination. These findings suggest that stigma is an overlooked but important aspect of IBD. Increasing awareness and offering psychological or social support may help patients cope better with their condition. Such approaches may also improve treatment adherence and reduce disease burden among younger patients. Future studies evaluating interventions aimed at reducing stigma and improving psychosocial well-being in patients with IBD may provide important clinical benefits. Longitudinal studies are also needed to clarify these relationships and to better understand how stigma affects the long-term course of IBD.

## Supporting information

S1 Dataset Anonymized dataset used for the analyses in the study. (ZIP)
