# Supplementary material for: More than a digestive disorder: Perceived stigma in inflammatory bowel disease patients
Source: PLoS One. 2026 Jun 26;21(6):e0352696. doi: 10.1371/journal.pone.0352696 (PMC13309050; doi:10.1371/journal.pone.0352696)
Supplement: Attachment — Submitted filename: Response to Reviewers.docx [file pone.0352696.s002.docx]

**Response to Editor**

**Dear Editor,**

Dear Dr. Martínez-Vázquez,

We sincerely thank you for your careful evaluation of our manuscript entitled “More than a digestive disorder: Perceived stigma in inflammatory bowel disease patients”. We are grateful for the constructive and insightful comments, which helped us improve the scientific clarity and methodological rigor of our study.

All comments and suggestions have been carefully addressed. The revisions made in the manuscript have been highlighted in the revised document, and a detailed point-by-point response is provided below.

We believe that these revisions have improved the clarity, methodological rigor, and overall quality of the manuscript. We sincerely thank the reviewers for their insightful suggestions and hope that the revised version will now be suitable for publication in PLOS One.

Sincerely

EC: Editor Comment

REC: Response to Editor Comment

**EC1.** Please ensure that your manuscript meets PLOS ONE's style requirements, including those for file naming. The PLOS ONE style templates can be found at

<https://journals.plos.org/plosone/s/file?id=wjVg/PLOSOne_formatting_sample_main_body.pdf> and

[https://journals.plos.org/plosone/s/file?id=ba62/PLOSOne_formatting_sample_title_authors_affiliations.pdf](https://track.editorialmanager.com/CL0/https:%2F%2Fjournals.plos.org%2Fplosone%2Fs%2Ffile%3Fid=ba62%2FPLOSOne_formatting_sample_title_authors_affiliations.pdf/1/010f019d9920ff84-f83ff963-76ec-4e73-af0c-76a3e3741e9b-000000/MscyCg5emHJWuU91ZiK5xgdwp2_Uw4tkRHS6aHwbDiI=258)

**REC1.** Thank you for this comment. We carefully revised the manuscript according to the PLOS ONE formatting and file naming requirements. The manuscript structure, headings, figure and table formatting, references, and file names were updated in line with the provided PLOS ONE style templates.

**EC2.** We note that you have indicated that there are restrictions to data sharing for this study. For studies involving human research participant data or other sensitive data, we encourage authors to share de-identified or anonymized data. However, when data cannot be publicly shared for ethical reasons, we allow authors to make their data sets available upon request. For information on unacceptable data access restrictions, please see <http://journals.plos.org/plosone/s/data-availability#loc-unacceptable-data-access-restrictions>.

Before we proceed with your manuscript, please address the following prompts:

a) If there are ethical or legal restrictions on sharing a de-identified data set, please explain them in detail (e.g., data contain potentially identifying or sensitive patient information, data are owned by a third-party organization, etc.) and who has imposed them (e.g., a Research Ethics Committee or Institutional Review Board, etc.). Please also provide contact information for a data access committee, ethics committee, or other institutional body to which data requests may be sent.

b) If there are no restrictions, please upload the minimal anonymized data set necessary to replicate your study findings to a stable, public repository and provide us with the relevant URLs, DOIs, or accession numbers. Please see <http://www.bmj.com/content/340/bmj.c181.long> for guidelines on how to de-identify and prepare clinical data for publication. For a list of recommended repositories, please see <https://journals.plos.org/plosone/s/recommended-repositories>. You also have the option of uploading the data as Supporting Information files, but we would recommend depositing data directly to a data repository if possible.

Please update your Data Availability statement in the submission form accordingly.

**REC2.** Thank you for this important comment. In response, we prepared and uploaded a fully anonymized and de-identified dataset containing the data necessary to replicate the analyses and findings reported in the manuscript. All direct identifiers, including participant names and other potentially identifiable personal information, were removed prior to data sharing.

The anonymized dataset has been uploaded as Supporting Information in both SPSS (.sav) and Microsoft Excel (.xlsx) formats under the title “S1 Dataset. Anonymized dataset used for the analyses in the study.”

The Data Availability Statement in both the submission system and the manuscript has been updated accordingly and now states that all relevant data underlying the findings are available within the manuscript and its Supporting Information files.

In addition, the Data Availability Statement in the submission form states:

"All relevant data underlying the findings of this study are provided within the manuscript and its Supporting Information files. The anonymized dataset used for the analyses is available as S1 Dataset."

**EC3.** Please include your full ethics statement in the ‘Methods’ section of your manuscript file. In your statement, please include the full name of the IRB or ethics committee who approved or waived your study, as well as whether or not you obtained informed written or verbal consent. If consent was waived for your study, please include this information in your statement as well.

**REC3.** Thank you for this important comment. In response, we revised the ethics statement in the Methods section to include the full name of the ethics committee and approval details (Page 5 Lines 99-101). Ethical approval for this study was obtained from the Elazığ Fethi Sekin City Hospital Non-Interventional Research Ethics Committee (Decision No: 2025/19-5; Date: 20 November 2025). Informed consent was obtained electronically from all participants before participation in the online survey.

**EC4.** Please upload a copy of Supporting Information Figures and Tables, which you refer to in your text on page 19.

**REC4.** Thank you for this comment. We realized that we had misunderstood the Supporting Information requirement in the initial submission. The tables (Tables 1–4) and figure (Fig 1) referred to in the manuscript are part of the main manuscript and not Supporting Information files. Therefore, the incorrect Supporting Information section has been removed from the revised manuscript. The figure file has also been uploaded separately in accordance with the PLOS ONE figure submission requirements. The Supporting Information section now includes only the supplementary material intended for public access, namely “S1 Dataset. Anonymized dataset used for the analyses in the study.”

**RC5.** If the reviewer comments include a recommendation to cite specific previously published works, please review and evaluate these publications to determine whether they are relevant and should be cited. There is no requirement to cite these works unless the editor has indicated otherwise.

**REC5.** Thank you for this comment. We carefully reviewed the reviewer comments.

**EC6.** Please review your reference list to ensure that it is complete and correct. If you have cited papers that have been retracted, please include the rationale for doing so in the manuscript text, or remove these references and replace them with relevant current references. Any changes to the reference list should be mentioned in the rebuttal letter that accompanies your revised manuscript. If you need to cite a retracted article, indicate the article’s retracted status in the References list and also include a citation and full reference for the retraction notice.

**REC6.** Thank you for this comment. We carefully reviewed and revised the reference list to ensure accuracy, completeness, and consistency with PLOS ONE reference formatting requirements.

**Additional Editor Comments:**
Dear Authors,
Thank you for your manuscript "MORE THAN A DİGESTİVE DİSORDER: PERCEİVED STİGMA İN INFLAMMATORY BOWEL DİSEASE PATİENTS Perceived Stigma in Inflammatory Bowel Disease", a study pertinent and relevant.
I found to aspects to solve:
- In the text, the authors mention about the high correlation among self- steem and stigma so, do they recommend to use any scale or how to discern to use one or another? It needs a better explanation in the text.

**Response:** Thank you for this valuable comment. We would like to clarify that we did not describe the relationship between stigma and self-esteem as a “high correlation” in the manuscript. Our findings only showed that patients reporting stigma had lower median RSES scores compared to those without stigma.

We would like to clarify that the present study did not aim to develop or validate a new stigma scale for inflammatory bowel disease. Instead, stigma was operationally defined as the presence of at least one “Yes” response to the ten disease-specific IBD stigma-related questions developed for this study based on previous literature and expert opinion. Therefore, no total stigma score or composite psychometric scale structure was created or analyzed.

The Rosenberg Self-Esteem Scale (RSES) was included to evaluate general self-esteem rather than disease-specific stigma. We agree that self-esteem and stigma are conceptually related but represent different psychosocial constructs. While the RSES measures overall self-worth, the IBD stigma-related questions used in this study specifically explored disease-related social experiences, behavioral avoidance, disclosure concerns, and perceived social judgment associated with IBD. For this reason, we believe that evaluating IBD-related stigma requires more disease-focused and context-specific questioning, as performed in our study.

In response to the editor’s suggestion, we have clarified this conceptual distinction more explicitly in the Discussion section. We also added a statement indicating that future studies may focus on the development and psychometric validation of a standardized IBD-specific stigma scale (Page 16 Lines 275-284).

**Additional Editor Comments:**

- In this context, I suggest to use a regression analysis including these scales.

**Response to Additional Editor Comments:**

Thank you for this valuable suggestion. In response to the editor’s recommendation, we performed a binary logistic regression analysis including variables that showed statistically significant differences between patients with and without perceived stigma in univariable analyses. Table 4 showed statistically significant differences between patients with and without perceived stigma in univariable analyses about age, age at diagnosis, and RSES total score. Patients with perceived stigma were significantly younger, had a younger age at diagnosis, and had lower RSES total scores compared to patients without perceived stigma. However, because age and age at diagnosis are closely related, we preferred to include only age in the regression model to avoid including two age-related variables simultaneously.

The regression analysis showed that younger age and lower RSES total scores were independently associated with perceived stigma. Since there is no universally accepted cutoff value for the RSES in the literature, both age and RSES total score were included in the model as continuous variables.

Because the regression model included only two variables, we did not add a separate regression table to the main manuscript in order to avoid unnecessary expansion of the Results section. However, the regression results are provided below for the editor’s evaluation (Page 14 Lines 221-232). In addition, the Statistical Analysis section of the Methods was revised to include a detailed description of the regression analysis procedure and reporting approach (Page 7 Lines 152-156). The Discussion section was also revised to discuss the independent association of younger age and lower self-esteem with perceived stigma and to clarify the conceptual distinction between general self-esteem and disease-specific stigma (Page 15 Lines 262-265; Page 16 Lines 272-275).

**Table. Binary logistic regression analysis for factors associated with perceived stigma**

| **Variables** | **B** | **OR** | **95% CI** | **p value** |
| --- | --- | --- | --- | --- |
| Age | -0.036 | 0.964 | 0.941-0.988 | 0.003 |
| RSES total score | -0.122 | 0.885 | 0.814-0.962 | 0.004 |

**Reviewers Comments to the Author

Reviewer 1’s Comment:** This study addresses a psychosocial dimension of IBD in a Turkish patient population, with a central finding linking perceived stigma to younger age and lower self-esteem. The manuscript may require revision addressing both the administrative and methodological shortcomings as stated below.

**Response:** We would like to thank Reviewer 1 for the careful evaluation of our manuscript and for the valuable and constructive comments. We have revised the manuscript accordingly and addressed all points raised. Our detailed, point-by-point responses are presented below.

R1C. Reviewer 1 Comment

RR1C. Response to Reviewer 1 Comment

**R1C1.** Ethics Statement is incomplete in Material and Methods section. The ethics approval number is explicitly left as "approval number to be inserted."

**RR1C1.** Thank you for this important comment. We revised the Ethics Statement in the Materials and Methods section to include the full ethics committee approval information (Page 5 Lines 99-101).

**R1C2.** The Methods section states that "the relationship between the scales' scores was assessed using Spearman's correlation," but the correlation results were not found in the Results.

**RR1C2.** Thank you for pointing this out. The sentence “The relationship between the scales’ scores was assessed using Spearman’s correlation” was included in the Statistical Analysis section by mistake. No correlation analysis was performed in this study. Therefore, this sentence has been removed from the revised manuscript to avoid confusion.

**R1C3.** The asterisk notation below Table 1 ("Bazı hastalarda birden fazka komplikasyon gözlenmiştir") is written in Turkish, not English.

**RR1C3.** Thank you for pointing this out. The Turkish sentence below Table 1 has been corrected and replaced with the English statement: “Some patients experienced more than one complication.”

**R1C4.** The absence of disease activity scores (e.g., Harvey-Bradshaw Index, Mayo Score) prevents taking symptom severity into account. And the online survey format excludes patients without internet access or digital literacy, thus it may make this study biasing toward younger, more educated respondents.

**RR1C4.** Thank you for this important comment. We agree that the absence of standardized disease activity indices, such as the Harvey-Bradshaw Index or Mayo Score, is an important limitation of the present study. Because disease activity was not formally assessed, we could not evaluate the potential relationship between symptom severity and perceived stigma. It is possible that active symptoms may have influenced some stigma-related experiences reported by participants.

We also agree that the online survey design may have introduced selection bias toward younger individuals and participants with better internet access or digital literacy. In response to the reviewer’s suggestion, we added these points to the Limitations section of the Discussion (Page 18 Lines 319-325.

**R1C5.** "inflammatory IBD" (line 250) is redundant. Either it should be "IBD" or "inflammatory bowel disease."

**RR1C5.** Thank you for pointing this out. The redundant expression “inflammatory IBD” has been corrected in the revised manuscript and replaced with “IBD.”

**Reviewer 2’s Comment:** My only concern is that the issue of stigma in IBD is well documented in the literature. A follow up study would be to evaluate interventions to address the stigma. I thought the study was well done, thorough and with good statistical support.

**Response:** Thank you for your valuable comments. We agree that future studies evaluating interventions to reduce stigma in patients with IBD would be important. In line with your suggestion, we revised the Conclusion section to emphasize the potential role of psychosocial support and future intervention-based studies, especially for younger patients and individuals with lower self-esteem (Page 18 Lines 328-337). We also thank you for your positive evaluation of our study.
