# Supplementary material for: More than a digestive disorder: Perceived stigma in inflammatory bowel disease patients
Source: PLoS One. 2026 Jun 26;21(6):e0352696. doi: 10.1371/journal.pone.0352696 (PMC13309050; doi:10.1371/journal.pone.0352696)
Supplement: Peer review history

# Peer review: Decision Letter 0

**DOI:** 10.1371/journal.pone.0352696.r001

**Authors:** Sophia Eugenia Martínez-Vázquez

16 Apr 2026

PONE-D-26-05994

MORE THAN A DİGESTİVE DİSORDER: PERCEİVED STİGMA İN INFLAMMATORY BOWEL DİSEASE PATİENTS Perceived Stigma in Inflammatory Bowel Disease

PLOS One

Dear Dr. Gunes Aydemır,

Thank you for submitting your manuscript to PLOS ONE. After careful consideration, we feel that it has merit but does not fully meet PLOS ONE’s publication criteria as it currently stands. Therefore, we invite you to submit a revised version of the manuscript that addresses the points raised during the review process.

Please submit your revised manuscript by May 31 2026 11:59PM. If you will need more time than this to complete your revisions, please reply to this message or contact the journal office at plosone@plos.org. When you're ready to submit your revision, log on to https://www.editorialmanager.com/pone/ and select the 'Submissions Needing Revision' folder to locate your manuscript file.

Please include the following items when submitting your revised manuscript:

- A letter that responds to each point raised by the academic editor and reviewer(s). You should upload this letter as a separate file labeled 'Response to Reviewers'.
- A marked-up copy of your manuscript that highlights changes made to the original version. You should upload this as a separate file labeled 'Revised Manuscript with Track Changes'.
- An unmarked version of your revised paper without tracked changes. You should upload this as a separate file labeled 'Manuscript'.

If you would like to make changes to your financial disclosure, please include your updated statement in your cover letter. Guidelines for resubmitting your figure files are available below the reviewer comments at the end of this letter.

If applicable, we recommend that you deposit your laboratory protocols in protocols.io to enhance the reproducibility of your results. Protocols.io assigns your protocol its own identifier (DOI) so that it can be cited independently in the future. For instructions see: https://journals.plos.org/plosone/s/submission-guidelines#loc-laboratory-protocols. Additionally, PLOS ONE offers an option for publishing peer-reviewed Lab Protocol articles, which describe protocols hosted on protocols.io. Read more information on sharing protocols at https://plos.org/protocols?utm_medium=editorial-email&utm_source=authorletters&utm_campaign=protocols.

As the corresponding author, your ORCID iD is verified in the submission system and will appear in the published article. PLOS supports the use of ORCID, and we encourage all coauthors to register for an ORCID iD and use it as well. Please encourage your coauthors to verify their ORCID iD within the submission system before final acceptance, as unverified ORCID iDs will not appear in the published article. *Only*  the individual author can complete the verification step; PLOS staff *cannot*  verify ORCID iDs on behalf of authors.

We look forward to receiving your revised manuscript.

Kind regards,

Sophia Eugenia Martínez-Vázquez, Ph.D.

Academic Editor

PLOS One

Journal Requirements:

When submitting your revision, we need you to address these additional requirements.

1. Please ensure that your manuscript meets PLOS ONE's style requirements, including those for file naming. The PLOS ONE style templates can be found at

https://journals.plos.org/plosone/s/file?id=wjVg/PLOSOne_formatting_sample_main_body.pdf and

https://journals.plos.org/plosone/s/file?id=ba62/PLOSOne_formatting_sample_title_authors_affiliations.pdf

2. We note that you have indicated that there are restrictions to data sharing for this study. For studies involving human research participant data or other sensitive data, we encourage authors to share de-identified or anonymized data. However, when data cannot be publicly shared for ethical reasons, we allow authors to make their data sets available upon request. For information on unacceptable data access restrictions, please see http://journals.plos.org/plosone/s/data-availability#loc-unacceptable-data-access-restrictions.

Before we proceed with your manuscript, please address the following prompts:

a) If there are ethical or legal restrictions on sharing a de-identified data set, please explain them in detail (e.g., data contain potentially identifying or sensitive patient information, data are owned by a third-party organization, etc.) and who has imposed them (e.g., a Research Ethics Committee or Institutional Review Board, etc.). Please also provide contact information for a data access committee, ethics committee, or other institutional body to which data requests may be sent.

b) If there are no restrictions, please upload the minimal anonymized data set necessary to replicate your study findings to a stable, public repository and provide us with the relevant URLs, DOIs, or accession numbers. Please see http://www.bmj.com/content/340/bmj.c181.long for guidelines on how to de-identify and prepare clinical data for publication. For a list of recommended repositories, please see https://journals.plos.org/plosone/s/recommended-repositories. You also have the option of uploading the data as Supporting Information files, but we would recommend depositing data directly to a data repository if possible.

Please update your Data Availability statement in the submission form accordingly.

3. Please include your full ethics statement in the ‘Methods’ section of your manuscript file. In your statement, please include the full name of the IRB or ethics committee who approved or waived your study, as well as whether or not you obtained informed written or verbal consent. If consent was waived for your study, please include this information in your statement as well.

4. Please upload a copy of Supporting Information Figures and Tables, which you refer to in your text on page 19.

5. If the reviewer comments include a recommendation to cite specific previously published works, please review and evaluate these publications to determine whether they are relevant and should be cited. There is no requirement to cite these works unless the editor has indicated otherwise.

6. Please review your reference list to ensure that it is complete and correct. If you have cited papers that have been retracted, please include the rationale for doing so in the manuscript text, or remove these references and replace them with relevant current references. Any changes to the reference list should be mentioned in the rebuttal letter that accompanies your revised manuscript. If you need to cite a retracted article, indicate the article’s retracted status in the References list and also include a citation and full reference for the retraction notice.

Additional Editor Comments:

Dear Authors,

Thank you for your manuscript "MORE THAN A DİGESTİVE DİSORDER: PERCEİVED STİGMA İN INFLAMMATORY BOWEL DİSEASE PATİENTS Perceived Stigma in Inflammatory Bowel Disease", a study pertinent and relevant.

I found to aspects to solve:

- In the text, the authors mention about the high correlation among self- steem and stigma so, do they recommend to use any scale or how to discern to use one or another? It needs a better explanation in the text.

- In this context, I suggest to use a regression analysis including these scales.

[Note: HTML markup is below. Please do not edit.]

Reviewers' comments:

Reviewer's Responses to Questions

**Comments to the Author**

1. Is the manuscript technically sound, and do the data support the conclusions?

The manuscript must describe a technically sound piece of scientific research with data that supports the conclusions. Experiments must have been conducted rigorously, with appropriate controls, replication, and sample sizes. The conclusions must be drawn appropriately based on the data presented.

Reviewer #1: Yes

Reviewer #2: Yes

**********

2. Has the statistical analysis been performed appropriately and rigorously?

Reviewer #1: I Don't Know

Reviewer #2: I Don't Know

**********

3. Have the authors made all data underlying the findings in their manuscript fully available?

The PLOS Data policy requires authors to make all data underlying the findings described in their manuscript fully available without restriction, with rare exception (please refer to the Data Availability Statement in the manuscript PDF file). The data should be provided as part of the manuscript or its supporting information, or deposited to a public repository. For example, in addition to summary statistics, the data points behind means, medians and variance measures should be available. If there are restrictions on publicly sharing data—e.g. participant privacy or use of data from a third party—those must be specified.

Reviewer #1: No

Reviewer #2: Yes

**********

4. Is the manuscript presented in an intelligible fashion and written in standard English?

PLOS ONE does not copyedit accepted manuscripts, so the language in submitted articles must be clear, correct, and unambiguous. Any typographical or grammatical errors should be corrected at revision, so please note any specific errors here.

Reviewer #1: Yes

Reviewer #2: Yes

**********

5. Review Comments to the Author

Please use the space provided to explain your answers to the questions above. You may also include additional comments for the author, including concerns about dual publication, research ethics, or publication ethics. (Please upload your review as an attachment if it exceeds 20,000 characters)

Reviewer #1: This study addresses a psychosocial dimension of IBD in a Turkish patient population, with a central finding linking perceived stigma to younger age and lower self-esteem. The manuscript may require revision addressing both the administrative and methodological shortcomings as stated below.

1) Ethics Statement is incomplete in Material and Methods section. The ethics approval number is explicitly left as "approval number to be inserted."

2) The Methods section states that "the relationship between the scales' scores was assessed using Spearman's correlation," but the correlation results were not found in the Results.

3) The asterisk notation below Table 1 ("Bazı hastalarda birden fazka komplikasyon gözlenmiştir") is written in Turkish, not English.

4) The absence of disease activity scores (e.g., Harvey-Bradshaw Index, Mayo Score) prevents taking symptom severity into account. And the online survey format excludes patients without internet access or digital literacy, thus it may make this study biasing toward younger, more educated respondents.

5) "inflammatory IBD" (line 250) is redundant. Either it should be "IBD" or "inflammatory bowel disease."

Reviewer #2: My only concern is that the issue of stigma in IBD is well documented in the literature. A follow up study would be to evaluate interventions to address the stigma. I thought the study was well done, thorough and with good statistical support.

**********

6. PLOS authors have the option to publish the peer review history of their article (what does this mean?). If published, this will include your full peer review and any attached files.

If you choose “no”, your identity will remain anonymous but your review may still be made public.

**Do you want your identity to be public for this peer review?**  For information about this choice, including consent withdrawal, please see our Privacy Policy.

Reviewer #1: No

Reviewer #2: No

**********

[NOTE: If reviewer comments were submitted as an attachment file, they will be attached to this email and accessible via the submission site. Please log into your account, locate the manuscript record, and check for the action link "View Attachments". If this link does not appear, there are no attachment files.]

To ensure your figures meet our technical requirements, please review our figure guidelines: https://journals.plos.org/plosone/s/figures

You may also use PLOS’s free figure tool, NAAS, to help you prepare publication quality figures: https://journals.plos.org/plosone/s/figures#loc-tools-for-figure-preparation.

NAAS will assess whether your figures meet our technical requirements by comparing each figure against our figure specifications.

# Author comment: Author response to Decision Letter 1

**DOI:** 10.1371/journal.pone.0352696.r002

9 Jun 2026

Response to Editor

Dear Editor,

Dear Dr. Martínez-Vázquez,

We sincerely thank you for your careful evaluation of our manuscript entitled “More than a digestive disorder: Perceived stigma in inflammatory bowel disease patients”. We are grateful for the constructive and insightful comments, which helped us improve the scientific clarity and methodological rigor of our study.

All comments and suggestions have been carefully addressed. The revisions made in the manuscript have been highlighted in the revised document, and a detailed point-by-point response is provided below.

We believe that these revisions have improved the clarity, methodological rigor, and overall quality of the manuscript. We sincerely thank the reviewers for their insightful suggestions and hope that the revised version will now be suitable for publication in PLOS One.

Sincerely

EC: Editor Comment

REC: Response to Editor Comment

EC1. Please ensure that your manuscript meets PLOS ONE's style requirements, including those for file naming. The PLOS ONE style templates can be found at

https://journals.plos.org/plosone/s/file?id=wjVg/PLOSOne_formatting_sample_main_body.pdf and

https://journals.plos.org/plosone/s/file?id=ba62/PLOSOne_formatting_sample_title_authors_affiliations.pdf

REC1. Thank you for this comment. We carefully revised the manuscript according to the PLOS ONE formatting and file naming requirements. The manuscript structure, headings, figure and table formatting, references, and file names were updated in line with the provided PLOS ONE style templates.

EC2. We note that you have indicated that there are restrictions to data sharing for this study. For studies involving human research participant data or other sensitive data, we encourage authors to share de-identified or anonymized data. However, when data cannot be publicly shared for ethical reasons, we allow authors to make their data sets available upon request. For information on unacceptable data access restrictions, please see http://journals.plos.org/plosone/s/data-availability#loc-unacceptable-data-access-restrictions.

Before we proceed with your manuscript, please address the following prompts:

a) If there are ethical or legal restrictions on sharing a de-identified data set, please explain them in detail (e.g., data contain potentially identifying or sensitive patient information, data are owned by a third-party organization, etc.) and who has imposed them (e.g., a Research Ethics Committee or Institutional Review Board, etc.). Please also provide contact information for a data access committee, ethics committee, or other institutional body to which data requests may be sent.

b) If there are no restrictions, please upload the minimal anonymized data set necessary to replicate your study findings to a stable, public repository and provide us with the relevant URLs, DOIs, or accession numbers. Please see http://www.bmj.com/content/340/bmj.c181.long for guidelines on how to de-identify and prepare clinical data for publication. For a list of recommended repositories, please see https://journals.plos.org/plosone/s/recommended-repositories. You also have the option of uploading the data as Supporting Information files, but we would recommend depositing data directly to a data repository if possible.

Please update your Data Availability statement in the submission form accordingly.

REC2. Thank you for this important comment. In response, we prepared and uploaded a fully anonymized and de-identified dataset containing the data necessary to replicate the analyses and findings reported in the manuscript. All direct identifiers, including participant names and other potentially identifiable personal information, were removed prior to data sharing.

The anonymized dataset has been uploaded as Supporting Information in both SPSS (.sav) and Microsoft Excel (.xlsx) formats under the title “S1 Dataset. Anonymized dataset used for the analyses in the study.”

The Data Availability Statement in both the submission system and the manuscript has been updated accordingly and now states that all relevant data underlying the findings are available within the manuscript and its Supporting Information files.

In addition, the Data Availability Statement in the submission form states:

"All relevant data underlying the findings of this study are provided within the manuscript and its Supporting Information files. The anonymized dataset used for the analyses is available as S1 Dataset."

EC3. Please include your full ethics statement in the ‘Methods’ section of your manuscript file. In your statement, please include the full name of the IRB or ethics committee who approved or waived your study, as well as whether or not you obtained informed written or verbal consent. If consent was waived for your study, please include this information in your statement as well.

REC3. Thank you for this important comment. In response, we revised the ethics statement in the Methods section to include the full name of the ethics committee and approval details (Page 5 Lines 99-101). Ethical approval for this study was obtained from the Elazığ Fethi Sekin City Hospital Non-Interventional Research Ethics Committee (Decision No: 2025/19-5; Date: 20 November 2025). Informed consent was obtained electronically from all participants before participation in the online survey.

EC4. Please upload a copy of Supporting Information Figures and Tables, which you refer to in your text on page 19.

REC4. Thank you for this comment. We realized that we had misunderstood the Supporting Information requirement in the initial submission. The tables (Tables 1–4) and figure (Fig 1) referred to in the manuscript are part of the main manuscript and not Supporting Information files. Therefore, the incorrect Supporting Information section has been removed from the revised manuscript. The figure file has also been uploaded separately in accordance with the PLOS ONE figure submission requirements. The Supporting Information section now includes only the supplementary material intended for public access, namely “S1 Dataset. Anonymized dataset used for the analyses in the study.”

RC5. If the reviewer comments include a recommendation to cite specific previously published works, please review and evaluate these publications to determine whether they are relevant and should be cited. There is no requirement to cite these works unless the editor has indicated otherwise.

REC5. Thank you for this comment. We carefully reviewed the reviewer comments.

EC6. Please review your reference list to ensure that it is complete and correct. If you have cited papers that have been retracted, please include the rationale for doing so in the manuscript text, or remove these references and replace them with relevant current references. Any changes to the reference list should be mentioned in the rebuttal letter that accompanies your revised manuscript. If you need to cite a retracted article, indicate the article’s retracted status in the References list and also include a citation and full reference for the retraction notice.

REC6. Thank you for this comment. We carefully reviewed and revised the reference list to ensure accuracy, completeness, and consistency with PLOS ONE reference formatting requirements.

Additional Editor Comments:

Dear Authors,

Thank you for your manuscript "MORE THAN A DİGESTİVE DİSORDER: PERCEİVED STİGMA İN INFLAMMATORY BOWEL DİSEASE PATİENTS Perceived Stigma in Inflammatory Bowel Disease", a study pertinent and relevant.

I found to aspects to solve:

- In the text, the authors mention about the high correlation among self- steem and stigma so, do they recommend to use any scale or how to discern to use one or another? It needs a better explanation in the text.

Response: Thank you for this valuable comment. We would like to clarify that we did not describe the relationship between stigma and self-esteem as a “high correlation” in the manuscript. Our findings only showed that patients reporting stigma had lower median RSES scores compared to those without stigma.

We would like to clarify that the present study did not aim to develop or validate a new stigma scale for inflammatory bowel disease. Instead, stigma was operationally defined as the presence of at least one “Yes” response to the ten disease-specific IBD stigma-related questions developed for this study based on previous literature and expert opinion. Therefore, no total stigma score or composite psychometric scale structure was created or analyzed.

The Rosenberg Self-Esteem Scale (RSES) was included to evaluate general self-esteem rather than disease-specific stigma. We agree that self-esteem and stigma are conceptually related but represent different psychosocial constructs. While the RSES measures overall self-worth, the IBD stigma-related questions used in this study specifically explored disease-related social experiences, behavioral avoidance, disclosure concerns, and perceived social judgment associated with IBD. For this reason, we believe that evaluating IBD-related stigma requires more disease-focused and context-specific questioning, as performed in our study.

In response to the editor’s suggestion, we have clarified this conceptual distinction more explicitly in the Discussion section. We also added a statement indicating that future studies may focus on the development and psychometric validation of a standardized IBD-specific stigma scale (Page 16 Lines 275-284).

Additional Editor Comments:

- In this context, I suggest to use a regression analysis including these scales.

Response to Additional Editor Comments:

Thank you for this valuable suggestion. In response to the editor’s recommendation, we performed a binary logistic regression analysis including variables that showed statistically significant differences between patients with and without perceived stigma in univariable analyses. Table 4 showed statistically significant differences between patients with and without perceived stigma in univariable analyses about age, age at diagnosis, and RSES total score. Patients with perceived stigma were significantly younger, had a younger age at diagnosis, and had lower RSES total scores compared to patients without perceived stigma. However, because age and age at diagnosis are closely related, we preferred to include only age in the regression model to avoid including two age-related variables simultaneously.

The regression analysis showed that younger age and lower RSES total scores were independently associated with perceived stigma. Since there is no universally accepted cutoff value for the RSES in the literature, both age and RSES total score were included in the model as continuous variables.

Because the regression model included only two variables, we did not add a separate regression table to the main manuscript in order to avoid unnecessary expansion of the Results section. However, the regression results are provided below for the editor’s evaluation (Page 14 Lines 221-232). In addition, the Statistical Analysis section of the Methods was revised to include a detailed description of the regression analysis procedure and reporting approach (Page 7 Lines 152-156). The Discussion section was also revised to discuss the independent association of younger age and lower self-esteem with perceived stigma and to clarify the conceptual distinction between general self-esteem and disease-specific stigma (Page 15 Lines 262-265; Page 16 Lines 272-275).

Table. Binary logistic regression analysis for factors associated with perceived stigma

Variables B OR 95% C p value

Age -0.036 0.964 0.941-0.988 0.003

RSES total score -0.122 0.885 0.814-0.962 0.004

________________________________________

Reviewers Comments to the Author

Reviewer 1’s Comment: This study addresses a psychosocial dimension of IBD in a Turkish patient population, with a central finding linking perceived stigma to younger age and lower self-esteem. The manuscript may require revision addressing both the administrative and methodological shortcomings as stated below.

Response: We would like to thank Reviewer 1 for the careful evaluation of our manuscript and for the valuable and constructive comments. We have revised the manuscript accordingly and addressed all points raised. Our detailed, point-by-point responses are presented below.

R1C. Reviewer 1 Comment

RR1C. Response to Reviewer 1 Comment

R1C1. Ethics Statement is incomplete in Material and Methods section. The ethics approval number is explicitly left as "approval number to be inserted."

RR1C1. Thank you for this important comment. We revised the Ethics Statement in the Materials and Methods section to include the full ethics committee approval information (Page 5 Lines 99-101).

R1C2. The Methods section states that "the relationship between the scales' scores was assessed using Spearman's correlation," but the correlation results were not found in the Results.

RR1C2. Thank you for pointing this out. The sentence “The relationship between the scales’ scores was assessed using Spearman’s correlation” was included in the Statistical Analysis section by mistake. No correlation analysis was performed in this study. Therefore, this sentence has been removed from the revised manuscript to avoid confusion.

R1C3. The asterisk notation below Table 1 ("Bazı hastalarda birden fazka komplikasyon gözlenmiştir") is written in Turkish, not English.

RR1C3. Thank you for pointing this out. The Turkish sentence below Table 1 has been corrected and replaced with the English statement: “Some patients experienced more than one complication.”

R1C4. The absence of disease activity scores (e.g., Harvey-Bradshaw Index, Mayo Score) prevents taking symptom severity into account. And the online survey format excludes patients without internet access or digital literacy, thus it may make this study biasing toward younger, more educated respondents.

RR1C4. Thank you for this important comment. We agree that the absence of standardized disease activity indices, such as the Harvey-Bradshaw Index or Mayo Score, is an important limitation of the present study. Because disease activity was not formally assessed, we could not evaluate the potential relationship between symptom severity and perceived stigma. It is possible that active symptoms may have influenced some stigma-related experiences reported by participants.

We also agree that the online survey design may have introduced selection bias toward younger individuals and participants with better internet access or digital literacy. In response to the reviewer’s suggestion, we added these points to the Limitations section of the Discussion (Page 18 Lines 319-325.

R1C5. "inflammatory IBD" (line 250) is redundant. Either it should be "IBD" or "inflammatory bowel disease."

RR1C5. Thank you for pointing this out. The redundant expression “inflammatory IBD” has been corrected in the revised manuscript and replaced with “IBD.”

________________________________________

Reviewer 2’s Comment: My only concern is that the issue of stigma in IBD is well documented in the literature. A follow up study would be to evaluate interventions to address the stigma. I thought the study was well done, thorough and with good statistical support.

Response: Thank you for your valuable comments. We agree that future studies evaluating interventions to reduce stigma in patients with IBD would be important. In line with your suggestion, we revised the Conclusion section to emphasize the potential role of psychosocial support and future intervention-based studies, especially for younger patients and individuals with lower self-esteem (Page 18 Lines 328-337). We also thank you for your positive evaluation of our study.

________________________________________

Attachment Submitted filename: Response to Reviewers.docx

# Editor report: Decision Letter 1

**DOI:** 10.1371/journal.pone.0352696.r003

**Authors:** Sophia Eugenia Martínez-Vázquez

14 Jun 2026

More than a digestive disorder: Perceived stigma in inflammatory bowel disease patients

PONE-D-26-05994R1

Dear Dr. Neslihan Gunes Aydemır

We’re pleased to inform you that your manuscript has been judged scientifically suitable for publication and will be formally accepted for publication once it meets all outstanding technical requirements.

Within one week, you’ll receive an e-mail detailing the required amendments. When these have been addressed, you’ll receive a formal acceptance letter and your manuscript will be scheduled for publication.

An invoice will be generated when your article is formally accepted. Please note, if your institution has a publishing partnership with PLOS and your article meets the relevant criteria, all or part of your publication costs will be covered. Please make sure your user information is up-to-date by logging into Editorial Manager at Editorial Manager® and clicking the ‘Update My Information' link at the top of the page. For questions related to billing, please contact billing support.

If your institution or institutions have a press office, please notify them about your upcoming paper to help maximize its impact. If they’ll be preparing press materials, please inform our press team as soon as possible -- no later than 48 hours after receiving the formal acceptance. Your manuscript will remain under strict press embargo until 2 pm Eastern Time on the date of publication. For more information, please contact onepress@plos.org.

Kind regards,

Sophia Eugenia Martínez-Vázquez, Ph.D.

Academic Editor

PLOS One

Additional Editor Comments (optional):

Dear Authors,

Your answers to all the comments satisfied the reviewers request. Thank you and congratulations.

Reviewers' comments:

# Editor report: Acceptance letter

**DOI:** 10.1371/journal.pone.0352696.r004

**Authors:** Sophia Eugenia Martínez-Vázquez

PONE-D-26-05994R1

PLOS One

Dear Dr. Güneş Aydemir,

I'm pleased to inform you that your manuscript has been deemed suitable for publication in PLOS One. Congratulations! Your manuscript is now being handed over to our production team.

At this stage, our production department will prepare your paper for publication. This includes ensuring the following:

* All references, tables, and figures are properly cited

* All relevant supporting information is included in the manuscript submission,

* There are no issues that prevent the paper from being properly typeset

You will receive further instructions from the production team, including instructions on how to review your proof when it is ready. Please keep in mind that we are working through a large volume of accepted articles, so please give us a few days to review your paper and let you know the next and final steps.

Lastly, if your institution or institutions have a press office, please let them know about your upcoming paper now to help maximize its impact. If they'll be preparing press materials, please inform our press team within the next 48 hours. Your manuscript will remain under strict press embargo until 2 pm Eastern Time on the date of publication. For more information, please contact onepress@plos.org.

You will receive an invoice from PLOS for your publication fee after your manuscript has reached the completed accept phase. If you receive an email requesting payment before acceptance or for any other service, this may be a phishing scheme. Learn how to identify phishing emails and protect your accounts at https://explore.plos.org/phishing.

If we can help with anything else, please email us at customercare@plos.org.

Thank you for submitting your work to PLOS ONE and supporting open access.

Kind regards,

PLOS ONE Editorial Office Staff

on behalf of

Dr. Sophia Eugenia Martínez-Vázquez

Academic Editor

PLOS One
